# Hereditary protein S deficiency leads to ischemic stroke

**DOI:** 10.3892/mmr.2015.3793

**Published:** 2015-05-18

**Authors:** ZHAO-HUI WANG, ZHI-JUN ZHAO, KANG XU, GUO-BING SUN, LIN SONG, HONG-XIANG YIN, XIAO-QI CHEN

**Affiliations:** 1 Department of Neurology, Hanyang Hospital, Wuhan University of Science and Technology, Wuhan, Hubei 430050, P.R. China; 2 Department of Ultrasonic Diagnosis and Interventional Therapy, The First Hospital Affiliated to Henan University, Kaifeng, Henan 475001, P.R. China; 3 Department of Neurology, Xinhua Hospital of Hubei, Wuhan, Hubei 430015, P.R. China

**Keywords:** protein S, mutation, expression study, arterial thrombotic disease

## Abstract

Hereditary protein S (PS) deficiency is an independent risk factor for venous thromboembolism. However, the correlation between PS and arterial thrombotic disease, such as cerebral thrombosis, is not clear. The present study focused on the molecular mechanisms underlying ischemic stroke caused by a PS gene mutation in one family. The activity of antithrombin, protein C and PS in the plasma of the proband was measured, and the genes encoding PS were amplified and sequenced. The cellular localization and expression of PS were analyzed in HEK-293 cells. The proband was a 50-year-old male. Plasma PS activity of the proband was 38.9%, which was significantly decreased compared with normal levels. Sequencing analysis revealed a PROS1 c.1486_1490delGATTA mutation on exon 12. This frameshift mutation converts Asp496 in the precursor PS into the termination codon. In addition, the PROS1 mutation was correlated with low PS activity in the family. Functional tests revealed that the mutant protein aggregated in the cytoplasm and its secretion and expression decreased. In conclusion, protein S mutation appeared to be the primary cause of thrombosis in the family of the present study. However, the correlation between PS deficiency and ischemic stroke requires further investigation.

## Introduction

Protein S (PS) is a single-chain, vitamin K-dependent glycoprotein located in the plasma, which is important in the protein C anticoagulant system (1). In the anticoagulation process that regulates thrombin generation, PS is an important cofactor for activated protein C to deactivate coagulation factor V and factor VIII (2). In addition, a recent study revealed that PS is also a significant cofactor for the tissue factor pathway inhibitor to inhibit blood coagulation factor X (3). Therefore, PS deficiency is an important cause of thrombophilia. Hereditary PS deficiency (PSD) is an independent risk factor of venous thromboembolism (4); however, whether it is a risk factor for ischemic stroke remains controversial (5–7). Although previous studies have confirmed an association between PS deficiency and ischemic stroke venous thrombosis (8,9), there is little reported evidence regarding ischemic stroke caused by PSD (10,11). Epidemiological studies have shown a higher risk of ischemic stroke in patients with unexplained venous thromboembolism than the average population, which suggests that venous and arterial thrombosis are caused by a common pathological process (12). In addition to anticoagulative effects, PS exhibits anti-inflammatory and anti-apoptotic effects (13–16). In a mouse model of ischemic stroke, injection of PS significantly reduced the size and extent of the infarct (17). In the present study, a pedigree of hereditary PSD was investigated with respect to ischemic stroke as well as the potential underlying molecular mechanisms.

## Materials and methods

### Pedigree

**Figure 1:**
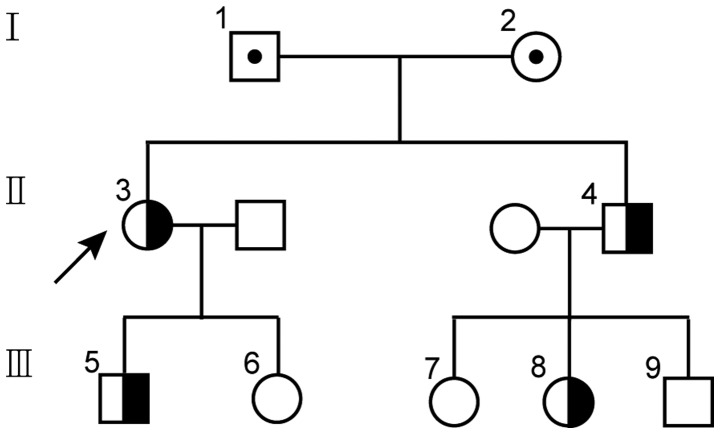
Family Tree: Males and females are shown as squares and circles, respectively. Half solid symbols represet individuals heterozygous for the PS mutation. The arrow indicates the proband.

A family with hereditary PSD was recruited into the study, which consisted of nine patients from three generations (Fig. 1). Research approval was obtained from the ethics committee of Xinhua Hospital of Hubei (Wuhan, China). Participants provided written informed consent in order to participate in this study and for the publication of their details. Family members had no other acquired risk factors that can cause thrombosis, for example they were not smokers and did not exhibit hyperlipidemia. The proband was a 50 year-old male patient with a large area cerebral infarction, which occurred without any apparent cause. Blood tests revealed no other abnormalities; blood coagulation function (prothrombin time and activated partial thromboplastin time) was also in the normal range. The screen for thrombotic disease revealed no hereditary antithrombin or protein C deficiency, as well as no factor V Leiden mutation or factor II G20210A mutation (18).

### Molecular tests

Peripheral blood samples were anticoagulated using sodium citrate and collected. The specimens were then centrifuged for 20 min at a speed of 2,500 x g to isolate platelet-poor plasma. The plasma PS anticoagulant activity was measured using an Automatic Coagulation Analyzer kit (Diagnostica Stago, Asnieres, France). Total PS antigen and free PS antigen were detected using ELISA (Diagnostica Stago). Results that did not lie within the normal range (60–140 IU/dL) were retested to confirm.

### Magnetic resonance imaging (MRI) test

The stroke of the proband was confirmed by an MRI scan, the imaging diagnostic criteria and clinical diagnosis of which strictly comply with the guidelines for stroke diagnosis and treatment at home and abroad (19).

### PROS1 sequencing

Genomic DNA was extracted from leukocytes in the blood specimens using a centrifugal column method (Biotech, Beijing, China). Mutation of PROS1 was analyzed by polymerase chain reaction (PCR) and sequencing. The exon fragments of PROS1, their boundary regions and the promoter regions (20) were amplified, and the primers were employed according to a previous study (21). The sequencing of the amplified product was performed using an automated DNA sequencing analyzer (Applied Biosystems, Foster City, CA, USA), according to the manufacturer's instructions. The NCBI reference sequence of PROS1 was NM_000313.3. The results were analyzed using Chromas software 2.0 (Informer Technologies Inc. Foster City, CA, USA).

### Construction of vectors with mutated PS and cell transfection

Vectors containing wild-type PS cDNA were purchased from Shanghai Genechem Company (Genechem, Shanghai, China). The vector containing mutated PS was constructed by site-directed mutagenesis. Primer sequences for site-directed mutagenesis and the reaction conditions are shown in Table I. The PS vector sequence was confirmed to be accurate by bi-directional sequencing. HEK-293T cells were cultured under the recommended conditions and were transfected when the cell density reached 85% (22). Transfection was performed using Lipofectamine 2000 (Invitrogen Life Technologies, Carlsbad, CA, USA) in accordance with the conditions recommended in the specifications. HEK-293T cells (American Type Culture Collection, Manassas, VA, USA) were co-transfected with 4.0 *µ*g wild type (WT) plasmid, MT vector or empty vector as a control group and 1 *µ*g enhanced green fluorescent protein (EGFP) vector (GeneCopoeia, Rockville, MD, USA). Medium was changed to serum-free Dulbecco's modified Eagle's medium (Invitrogen Life Technologies) with vitamin K1 (Genechem Company) and antibiotics (penicillin and streptomycin; Invitrogen Life Technologies) 6 h after transient transfection. After 36 h, cell supernatants and transfected cell lines were generated for functional tests.

### PS expression in vitro

Total RNA was isolated from transfected cells using TRIzol (Invitrogen Life Technologies), and reverse transcription was performed via a Transcription kit (Invitrogen Life Technologies) to obtain cDNA. Using a Real-time PCR kit (Invitrogen Life Technologies), SYBR Green I was used for relative quantification of PS mRNA. Primer sequences used for real-time PCR are shown in Table I. EGFP was used to estimate the transfection efficiency. The experiment was repeated three times. Data were analyzed using unpaired t-tests. P<0.05 was considered to indicate a statistically significant difference.

The transfected cells were immunolabeled with Cy3-labeled mouse anti-human (cat. no. 16910-1-AP; Proteintech Group, Inc., Chicago, IL, USA). Laser confocal microscopy was performed on a Fluoview FV1000 laser-scanning confocal microscope (Olympus, Centre Valley, PA, USA), to determine the localization and expression of recombinant PS as described previously (23). Total protein of the transfected cells was determined by standard methods (24). Transfected cells were then incubated with horseradish peroxidase-labeled mouse anti-human antibody (cat. no. H00007791-M01; Genechem Company). Western blot analysis was performed to determine the quantity of intracellular PS. Protein concentration was measured using the BCA Protein Assay kit (Thermo Scientific, Waltham, MA, USA). The total cellular proteins were prepared by lysing transfected cells and then loading 40 *µ*g protein from each sample on 8% SDS-PAGE gels (Invitrogen Life Technologies). The proteins were transferred onto polyvinylidene fluoride membranes (Amersham Biosciences, Piscataway, NJ, USA). PS was detected using a goat polyclonal antibody against human ProS (1:1000; no. AF4036; R&D Systems, Minneapolis, MN, USA), followed by Rabbit Anti-Goat IgG horseraidish peroxidase-conjugated antibody (1:10,000; no. HAF017; R&D Systems). Primary antibodies were added in 5% milk in TBS-T (25 mM Tris, 140 mM NaCl, and 0.1% Tween 20, pH 7.5), and incubated on a shaker overnight at 4°C. The following morning, the membrane was washed three times for 10 min with 1 TBS-T. After the incubation with the secondary antibody, the membranes were washed three times for 10 min with 1 TBS-T. The signal was detected using enhanced chemilluminescence detection reagents (Amersham Biosciences) and results were obtained using a camera method on a Gel Doc EZ system (Bio-Rad Laboratories Ltd., Hertfordshire, UK).

The PS antigen level of cell culture supernatant was measured using ELISA (Stago) to determine the level of PS secretion. The results were standardized based on transfection efficiency. Each result was confirmed in three independent experiments. The groups were assessed statistically using non-parametric tests. The data are expressed as the mean ± standard deviation. The differences between the two groups were determined by Student's t-test using SPSS 13.0 softwared (SPSS, Inc., Chicago, IL, USA). P<0.05 was considered to indicate a statistically significant difference.

## Results

### PS level of family members and PROS1 mutation

**Figure 2:**
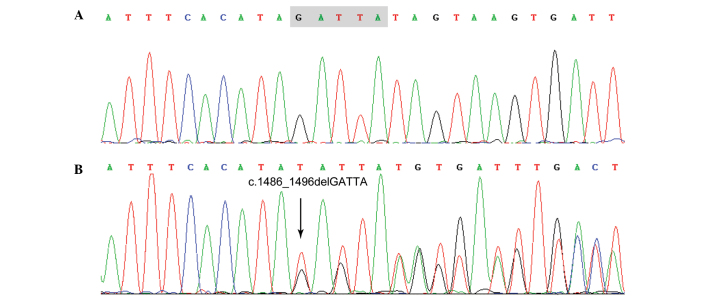
Sequencing diagram obtained using chromas software. (A) Result from a normal individual and (B) result from the proband with the PROS mutation.

The proband was confirmed repeatedly as exhibiting hereditary PSD type I. A novel deletion mutation, c.1486_1496delGATTA (p.Asp496^*^), was identified via mutation analysis of exon 12 of the PS gene of the proband, as shown in Fig. 2, which was verified by PCR and sequencing of an independent sample. The frame shift mutation resulted in the formation of a premature termination codon. Four family members were detected as PSD (Table II), with significantly reduced PS activity and antigen levels, and the same PS gene mutation.

### Functional tests of PS in vitro

**Figure 3:**
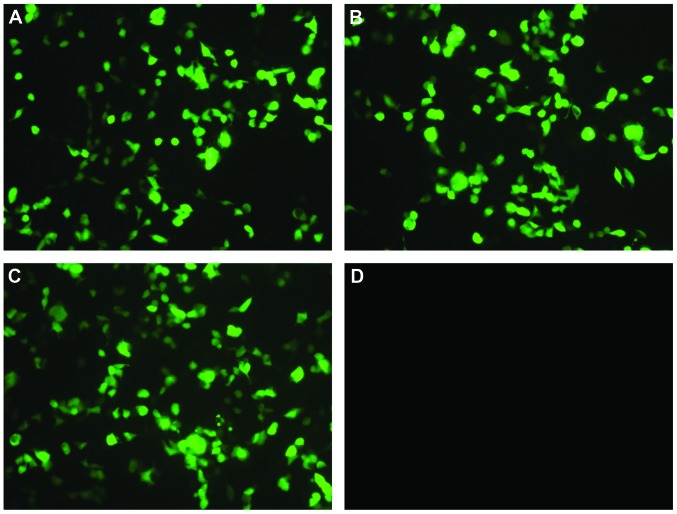
Cell transfection. The enhanced green flurorescence vector co-transfected with expression constructs, (A) wild type, (B) mutant, (C) empty vector and (D) blank control (magnification, x100).

**Figure 4:**
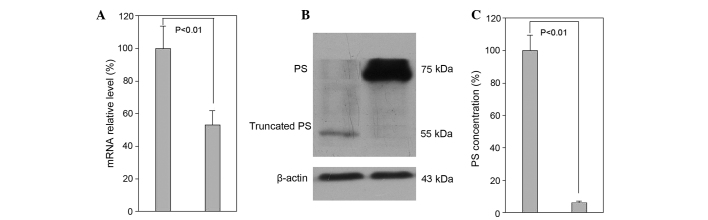
Recombinant PS expression experiments. (A) Relative quantification of mRNA levels by quantitative polymerase chain reaction. (B) Detection of intracellular antigens by western blot analysis using human protein S antibody. (C) Cell supernatant PS quantification by ELISA. PS, protein S.

RT-qPCR of cells transfected with recombinant PS expression vector (Fig. 3) revealed that the mRNA of MT PS contains only 53.1% of the complete WT mRNA. The PS band (~75 kDa) was present in the western blot analysis of the WT sample. However, a weak band at ~55 kDa was barely detectable in the MT sample, suggesting that the abnormal truncated PS mRNA resulted in unstable early termination (Fig. 4).

**Figure 5:**
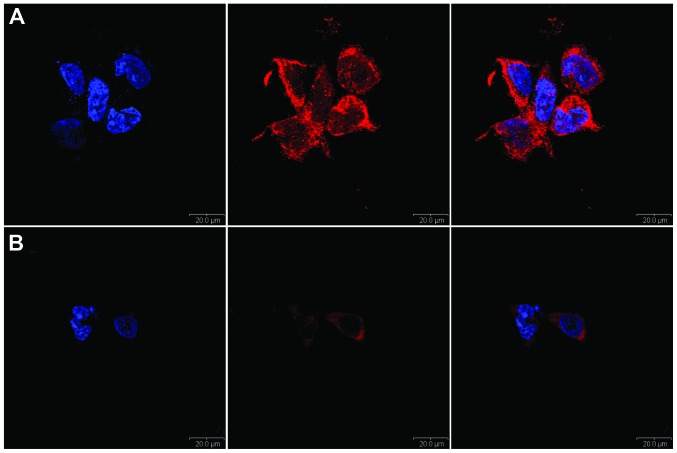
Indirect immunofluorescence and confocal laser scanning microscopy to determine the subcellular localization of recombinant PS. (A) Wild type PS and (B) mutant type PS. Scale bar, 200 *µ*m PS, protein S. Cell nuclei are shown in blue. Recombinant PS are displayed in red. Panels on the right are merged.

ELISA tests indicated that the quantity of MT PS in the cell supernatant was only 6.1% compared with that of the WT PS. Confocal laser microscopy (Fig. 5) showed that WT transfected cells expressed PS in the cytoplasm intensely and uniformly, and the fluorescence of MT PS was clearly decreased.

## Discussion

The PROS1 gene encoding PS, which spans ~80 kb of genomic DNA, is located on the 3q11.2 chromosome, and the mature PS protein Consists of 635 amino acids (25). In the plasma, 60% of the total PS forms a complex with C4b-binding protein, whereas 40% is unbound, and only free PS is active (26). The incidence rate of hereditary PSD (MIM 176880) in Europe and the United States is 0.03–0.13%. PSD is divided into three types: Type I PSD is characterized by the decrease in PS activity and the PS antigen; type II is characterized by reduced PS activity with a normal PS antigen; and type III is characterized by decreased PS activity, normal total PS antigen and decreased free PS antigen (27,28).

In the family investigated in the present study, a novel small deletion of the PS gene was observed. The mutation in the family members was consistent with their reduced PS level, indicating that this mutation was the molecular basis of the PSD and ischemic stroke. In addition, functional tests *in vitro* suggested that the mutant PS expression levels were significantly reduced, preventing its excretion into the culture media. Due to the nonsense-mediated mRNA degradation mechanism (29), the level of mutant PS mRNA was only 53.1% of the wild type mRNA transcription level, indicating that the mutant mRNA was unstable *in vivo*. At the protein expression level, mutant PS expression in the cell extract supernatants was greatly reduced, indicating that the mutant PS possessed an abnormal structure that was unstable and was degraded intracellularly, even though the truncated PS could be translated (30). Therefore, decreased PS expression is the basis of PSD in this family.

This study reported on the clinical and molecular analyses of a family with PSD. To the best of our knowledge, this is the first study to report this mutation in PS. The association between hereditary PSD and ischemic stroke requires further research and discussion.

## Figures and Tables

**Table I:** Primers for site-directed mutagenesis and real-time fluorescent quantitative polymerase chain reaction.

Primer	Primer sequence	Primer application	Annealing temperature (°C)
D-F	GCTCAATTTCACATATAATAATGTATCCAGTGCTGAGGG	Site-directed mutagenesis	68
D-R	CCCTCAGCACTGGATACATTATTATATGTGAAATTGAGC		
S-F	TGATTCAGAAGGCGTGATACT	Relative quantification	60
S-R	AGACACCATATTCCATAGACCATT		

D-F, detect primer forward; D-R, detect primer reverse; S-F, protein S primer forward; S-R, protein S primer reverse.

**Table II:** Genotype and clinical data of the family members.

Family member	Gender	Age (years)	p.Asp496^*^ mutation	PS activity (%)	Total PS antigen (%)	Free PS antigen (%)	Clinical manifestation
I- 1	M	82	ND	ND	ND	ND	Negative
I- 2	F	79	ND	ND	ND	ND	Negative
II- 3	F	52	Positive	41	58	52	Large area cerebral infarct
II- 4	M	50	Positive	37	46	50	Large area cerebral infarct
III- 5	M	25	Positive	32	50	35	Negative
III- 6	F	22	Negative	88	114	97	Negative
III- 7	F	26	Negative	85	80	123	Negative
III- 8	F	24	Positive	39	40	43	Negative
III- 9	M	21	Negative	80	73	83	Negative

Reference range: PS activity, 65–130%;total PS antigen, 70–130%; free PS antigen, 65–130%. M, male; F, female; ND, not determined.

## References

[1] Dahlback B, Villoutreix BO (2005). Regulation of blood coagulation by the protein C anticoagulant pathway: novel insights into structure-function relationships and molecular recognition. Arterioscler Thromb Vasc Biol.

[2] Tang L, Wang HF, Lu X (2013). Common genetic risk factors for venous thrombosis in the Chinese population. Am J Hum Genet.

[3] Hackeng TM, Sere KM, Tans G, Rosing J (2006). Protein S stimulates inhibition of the tissue factor pathway by tissue factor pathway inhibitor. Proc Natl Acad Sci USA.

[4] Ten Kate MK, Platteel M, Mulder R (2008). PROS1 analysis in 87 pedigrees with hereditary protein S deficiency demonstrates striking genotype-phenotype associations. Hum Mutat.

[5] Zlokovic BV, Zhang C, Liu D, Fernandez J, Griffin JH, Chopp M (2005). Functional recovery after embolic stroke in rodents by activated protein C. Ann Neurol.

[6] Guo H, Singh I, Wang Y (2009). Neuroprotective activities of activated protein C mutant with reduced anticoagulant activity. Eur J Neurosci.

[7] Soare AM, Popa C (2010). Deficiencies of proteins C, S and antithrombin and factor V Leiden and the risk of ischemic strokes. J Med Life.

[8] Sacco RL, Owen J, Mohr JP, Tatemichi TK, Grossman BA (1989). Free protein S deficiency: a possible association with cerebrovascular occlusion. Stroke.

[9] Barinagarrementeria F, Cantu-Brito C, De La Peña A, Izaguirre R (1994). Prothrombotic states in young people with idiopathic stroke. A prospective study Stroke.

[10] Kristensen B, Malm J, Carlberg B (1997). Epidemiology and etiology of ischemic stroke in young adults aged 18 to 44 years in northern Sweden. Stroke.

[11] Hankey GJ, Eikelboom JW, van Bockxmeer FM, Lofthouse E, Staples N, Baker RI (2001). Inherited thrombophilia in ischemic stroke and its pathogenic subtypes. Stroke.

[12] Sorensen HT, Horvath-Puho E, Pedersen L, Baron JA, Prandoni P (2007). Venous thromboembolism and subsequent hospitalisation due to acute arterial cardiovascular events: a 20-year cohort study. Lancet.

[13] Rezende SM, Simmonds RE, Lane DA (2004). Coagulation, inflammation and apoptosis: different roles for protein S and the protein S-C4b binding protein Complex. Blood.

[14] Loubele ST, Spek CA, Leenders P (2009). Activated protein C protects against myocardial ischemia/reperfusion injury via inhibition of apoptosis and inflammation. Arterioscler Thromb Vasc Biol.

[15] Cheng T, Liu D, Griffin JH (2003). Activated protein C blocks p53-mediated apoptosis in ischemic human brain endothelium and is neuroprotective. Nat Med.

[16] Fernandez JA, Xu X, Liu D, Zlokovic BV, Griffin JH (2003). Recombinant murine-activated protein C is neuroprotective in a murine ischemic stroke model. Blood Cells Mol Dis.

[17] Liu D, Guo H, Griffin JH, Fernandez JA, Zlokovic BV (2003). Protein S confers neuronal protection during ischemic/hypoxic injury in mice. Circulation.

[18] Dahlback B (2008). Advances in understanding pathogenic mechanisms of thrombophilic disorders. Blood.

[19] Adams HP, Bendixen BH, Kappelle LJ (1993). Classification of subtype of acute ischemic stroke. Definitions for use in a multicenter clinical trial. TOAST. Trial of Org 10172 in Acute Stroke Treatment. Stroke.

[20] Lundwall A, Dackowski W, Cohen E (1986). Isolation and sequence of the cDNA for human protein S, a regulator of blood coagulation. Proc Natl Acad Sci USA.

[21] Tang L, Lu X, Yu JM (2012). PROC c.574_576del polymorphism: a common genetic risk factor for venous thrombosis in the Chinese population. J Thromb Haemost.

[22] Okada H, Yamazaki T, Takagi A (2006). In vitro characterization of missense mutations associated with quantitative protein S deficiency. J Thromb Haemost.

[23] Rajgor D, Mellad JA, Autore F, Zhang Q, Shanahan CM (2012). Multiple novel nesprin-1 and nesprin-2 variants act as versatile tissue-specific intracellular scaffolds. PLoS One.

[24] Kralj JG1, Munson MS, Ross D (2014). Total protein quantitation using the bicinchoninic acid assay and gradient elution moving boundary electrophoresis. Electrophoresis.

[25] Edenbrandt CM, Lundwall A, Wydro R, Stenflo J (1990). Molecular analysis of the gene for vitamin K dependent protein S and its pseudogene. Cloning and partial gene organization Biochemistry.

[26] Maurissen LF, Thomassen MC, Nicolaes GA (2008). Re-evaluation of the role of the protein S-C4b binding protein Complex in activated protein C-catalyzed factor Va-inactivation. Blood.

[27] Dykes AC, Walker ID, McMahon AD, Islam SI, Tait RC (2001). A study of Protein S antigen levels in 3788 healthy volunteers: influence of age, sex and hormone use and estimate for prevalence of deficiency state. Br J Haematol.

[28] Biguzzi E, Razzari C, Lane DA (2005). Molecular diversity and thrombotic risk in protein S deficiency: the PROSIT study. Hum Mutat.

[29] Stalder L, Muhlemann O (2008). The meaning of nonsense. Trends Cell Biol.

[30] Singh G, Lykke-Andersen J (2003). New insights into the formation of active nonsense-mediated decay complexes. Trends Biochem Sci.

